# A Modern Approach to Minimally Invasive Surgery and Laparoscopic Sterilization in a Chimpanzee

**DOI:** 10.1155/2019/7492910

**Published:** 2019-09-23

**Authors:** Laura K. Newcomb, Meghan A. Kruse, Larry J. Minter, Craig J. Sobolewski

**Affiliations:** ^1^Department of Obstetrics and Gynecology, Division of Minimally Invasive Gynecologic Surgery, Duke University Medical Center, 200 Trent Drive, Durham, NC 27710, USA; ^2^Veterinary Specialty Hospital of the Carolinas, 6405 Tryon Rd, Cary, NC 27518, USA; ^3^North Carolina Zoo, 4401 Zoo Pkwy, Asheboro, NC 27205, USA

## Abstract

We present the case of Ruby, a 21-year-old hand-reared chimpanzee (*Pan troglodytes*) who had an obstetric history significant for a premature stillborn infant that was conceived while on oral contraceptive pills, followed by a full term healthy delivery complicated by neonatal demise attributed to inappropriate maternal care. She was recommended for permanent sterilization due to her history of conception while on oral contraceptives. She underwent uncomplicated laparoscopic bilateral tubal ligation. Due to the similar anatomy to humans, human OB/GYN surgical consultants were used. The objective of this case report is to describe a modern technique for approaching and employing laparoscopic surgery in primates. Minimally invasive surgery allows for faster recovery and fewer complications, and has become the preferred approach for surgical intervention in many animals. The information presented in this case report can be expanded to benefit not only Chimpanzees but other large primate species as well. However, subtle anatomical differences among species must be recognized in order to be carried out safely.

## 1. Introduction

The last published report of laparoscopic surgery in primates was in 1976 in the Journal of Medical Primatology by Charles Graham [1]. Laparoscopic surgery has become more common and surgical technology has improved since that time; however, there is no recent literature describing its techniques and benefits in primates. Given the close resemblance of size, anatomy, physiology, and disease pathology to humans, a modern technique for laparoscopic surgery in primates has been developed based on the standard human procedure and will be described here. The technique described here allows for not only laparoscopic pelvic organ surgery such as sterilization, oophorectomy, and hysterectomy but also offers an approach to a wider variety of intra-abdominal procedures including cholecystectomy, bowel surgery, urologic procedures, and more.

It is well established in the human medical literature that laparoscopic surgery has many benefits over traditional open surgery including shorter post-operative recovery periods, decreased risk of surgical complications, and decreased risk of wound infection [2]. The predominant trend in recent years has been to use laparoscopy as the default approach to intra-abdominal surgery in humans, and our hope is that this will carry over into the veterinary arena for primates as well. This report highlights key differences between the laparoscopic approach to primates and other more common animal species such as cats and dogs, and allows for a safe adoption of these practices by veterinarian laparoscopic surgeons.

## 2. Case Report

We present the case of Ruby, a 21-year-old female chimpanzee (*Pan troglodytes*) who was hand-reared at the North Carolina Zoo. Her first gestation was conceived while on oral contraceptive pills (1 mg norethindrone/50 mcg mestranol daily) and was complicated by a preterm delivery with a stillborn infant. Her second gestation was a full term livebirth; however, the infant died shortly thereafter. Her caretakers believed that the death was caused by inappropriate maternal care, specifically that the location where she carried her infant was inappropriate and lead to suffocation.

Behavioral development of all primates, human and nonhuman, has been demonstrated to be contingent upon early rearing experiences. In nonhuman primate social development, close bodily contact, whether by grooming, clinging, or play, are important components to this. Insufficient maternal care during development can also result in deficits in life skills and social development. In the captive environment, mitigating circumstances may result in separation of the infant from its mother, necessitating hand-rearing by human caregivers. Studies have found that hand-reared chimpanzees exhibit some stereotypical abnormal behaviors more frequently than mother-reared individuals, including self-clinging, body-rocking, and digit sucking, as well as a lack of social play, or inappropriate reactions to social situations that may persist into adulthood [3]. In this light, it is possible that Ruby's history of having been hand-reared may have impacted her maternal skills and her ability to care for her infant.

Due to Ruby's history, she was not going to be recommended for breeding again. Given that she had previously conceived while on oral contraceptives, the decision was made to proceed with permanent sterilization via laparoscopic bilateral tubal ligation. The procedure was performed at the Veterinary Specialty Hospital of the Carolinas in Cary, NC in February, 2018.

## 3. Selection of Equipment

A comprehensive list of equipment used is listed in Table 1. These laparoscopic instruments are largely similar in function from one brand to another, so no brand names were used. With the advent of “micro-laparoscopy”, one could consider substituting the 5 mm instruments used for these smaller 3 mm “micro-instruments” when available. This could further decrease incision sizes and complications such as a hernia or disruption of the wound by the chimp in the post-operative period.

When performing pelvic surgery, it is extremely helpful to have the ability to manipulate the uterus out of the pelvis and up into the abdomen for better visualization and access to the adnexa, vasculature, and other surrounding structures. While the use of a Hulka uterine manipulator was intended to achieve this goal, the cervical canal of this chimp (and perhaps many chimps) was small, stenotic, and difficult to access with the Hulka probe. It has been proposed to use a probe with a cuff that is inserted into the vagina and fits over the ectocervix, and can be secured on the cervix by suction. This was reportedly custom-made and it was not available for use, so instead a “sponge-stick” (a ring forcep with a surgical sponge wrapped and secured in the operative end of the forcep) was placed in the posterior fornix of the vagina, or dorsal to the cervix. When manipulated, the uterine body is moved opposite to the manipulation of the sponge stick.

## 4. Preparation of Subject

The patient was made NPO, or “nothing by mouth”, prior to the procedure. On arrival to the surgical center, she was pre-medicated with heavy sedation with no resistance, and given ketamine (500 mg IM), midazolam (10 mg IM), and medetomidine (5 mg IM). She was intubated with a 9 mm endotracheal tube and isoflurane was initiated and maintained through the procedure. A loading dose of lidocaine (50 mg IV) was given and the patient was transferred to the surgery preparation area. A catheter was placed in the saphenous vein. Once in the operating room, the patient's breathing circuit was connected to a Hallowell mechanical ventilator with respiratory rate and minute volume set to maintain ETCO_2_ between 35 and 45 mmHg. A loading dose of ketamine (25 mg IV) was given. A dose of buprenorphine (0.54 mg IV) was given for pain management. Lidocaine (20 mcg/kg/min) and ketamine (30 mcg/kg/min) were administered in a continuous rate infusion and adjusted as needed throughout surgery.

**Figure 1:**
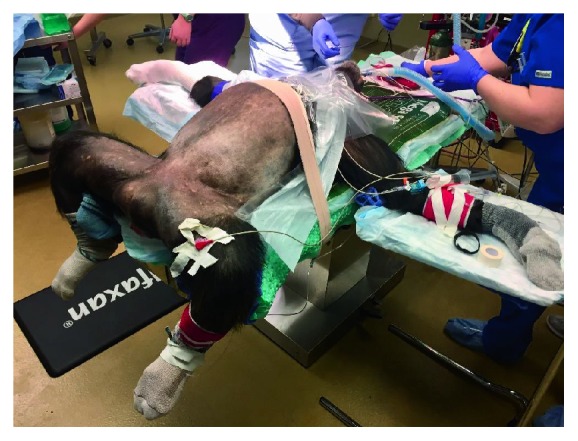
Positioning of the patient in a dorsal low lithotomy position.

With the animal in the dorsal recumbancy position, the entire abdomen was shaved and scrubbed. Shaving the entire abdomen in advance allows for easier conversion to laparotomy if needed. The animal was positioned at the end of the operating table, which allows for adequate access to the vagina and, therefore, manipulation of the uterus. The legs were secured to posts on the operating table and the arms were outstretched at an approximately 90 degree angle from the trunk, and secured on arm rests. The upper chest was secured to the table with tape to prevent the animal from sliding while in steep Trendelenburg position. See Figure 1 for positioning.

**Figure 2:**
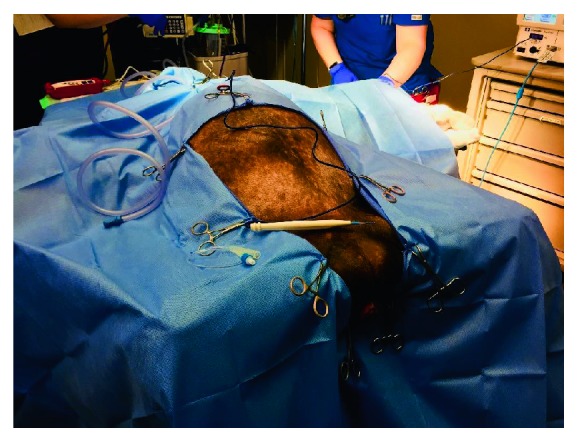
Draping of the patient.

Next the abdomen, perineum, and vagina were prepared for surgery using sterile soap and the patient was draped, leaving the abdomen and perineum exposed. See Figure 2 for draping. Next an attempt was made to drain the bladder by placing a foley catheter for continuous drainage during the operation; however, the anterior location of the urethra within the vaginal canal made access difficult. A smaller and larger size foley catheter were used to attempt placement without success. Instead, a suprapubic catheter was placed under direct laparoscopic visualization later in the case in order to drain the bladder.

## 5. Laparoscopy Technique

The laparoscope, light cables, insufflation tubing, and electrocautery cables were introduced into the field, assembled, and secured to the drapes. To gain entry to the abdominal cavity, the open Hasson technique was employed. A 5 mm semi-lunar incision was made inferior to the umbilicus. The subcutaneous fat was mobilized laterally until the fascia was exposed and grasped with two tonsil clamps. This was elevated and a scalpel was used to incise the fascia and gain entry to the peritoneal cavity. A 5 mm cannula was inserted and the 5 mm, 0 degree laparoscope was introduced to confirm intraperitoneal location. The insufflation tubing carrying carbon dioxide gas was connected to the cannula and the abdomen was insufflated to achieve pneumoperitoneum at a pressure of 7–8 mmHg.

The table was then tilted into Trendelenburg position to allow the bowel to move cranially in the abdomen. Lateral instrumental portals were then placed. To do this, the anterior abdominal wall was inspected for the location of the left and right inferior epigastric artery and vein, which typically run just lateral to their respective medial umbilical ligament. Care should be taken to ensure that instrumental portals are placed lateral to these vessels, as the origin of the inferior epigastric artery is the external iliac artery and a significant amount of bleeding can be encountered should it be transected. Of note, these vessels are not present in dogs, and this difference must be considered when planning port placement in primates. Next, a 5 mm skin incision was created with the scalpel, and a 5 mm instrumental portal was placed in both the left and the right lower quadrant with a penetrating trocar with cannula. The internal rectus fascia is notably thicker and tougher than in dogs and cats, and may require a penetrating port placement device.

Two 5 mm blunt grasping forceps were used to displace the bowel into the upper abdomen. This may not allow for full visualization of the pelvic organs, as some chimpanzees are found to have extensive abdominal adhesions despite no prior abdominal surgery. Elevation of the uterus with the manipulator may also help to bring the uterine fundus and adnexa into view. This is accomplished by pushing the handle of the manipulator inwards and down towards the perineum.

**Figure 3:**
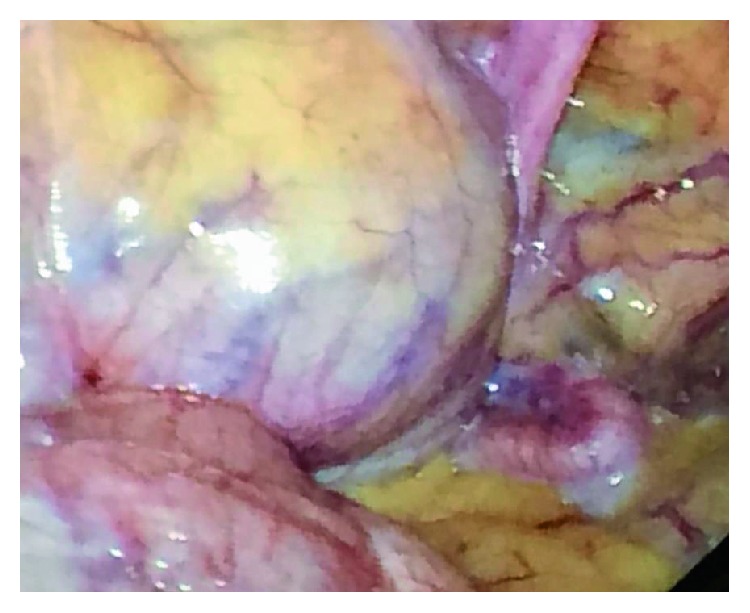
Intra-abdominal view of the pelvis. In the top left corner is the bladder, and the bottom left corner is the colon. The fallopian tube can be seen resting on the omentum in the bottom right.

Next the bipolar vessel sealing device was used to carefully take down adhesions of the omentum and colonic epiploica to the anterior abdominal wall. At this point, the bladder was identified; however, its anterior and cephalic position in the chimpanzee species made visualization of the operative field difficult (see Figure 3). The bladder is also an intraperitoneal structure in this species, which adds to its prominent location. At this stage, the decision was made to drain the bladder using a needle and a suprapubic catheter under direct visualization.

**Figure 4:**
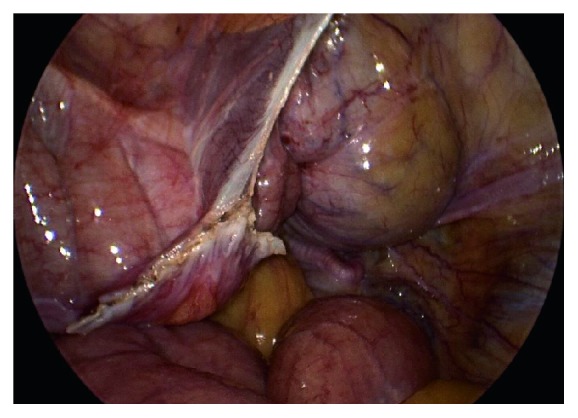
View of the pelvis following suprapubic drainage of the bladder, as well as the hemostatic site of lysis of adhesions (white), and colon on the left side.

The redundancy of the colon also inhibited full visualization of the uterus. In the chimpanzee species, the ascending and descending colon tend to lose their peritoneal supports, and the transverse mesocolon is long and well-developed and drapes into the surgical field [4]. Additionally, the haustra are very well developed and appear like large sacculations. These anatomical structures intruded into the operative field and obscured visualization. In order to move forward with the procedure and gain adequate visualization, a third 5 mm instrumental portal was placed in the abdominal midline just cranial to the cranial border of the bladder. A 5 mm fanning paddle retractor was inserted and used to successfully move the bowel laterally away from the operative field and protect the bowel from injury.  Figure 4 shows the pelvis after drainage of the bladder and displacement of the bowel.

**Figure 5:**
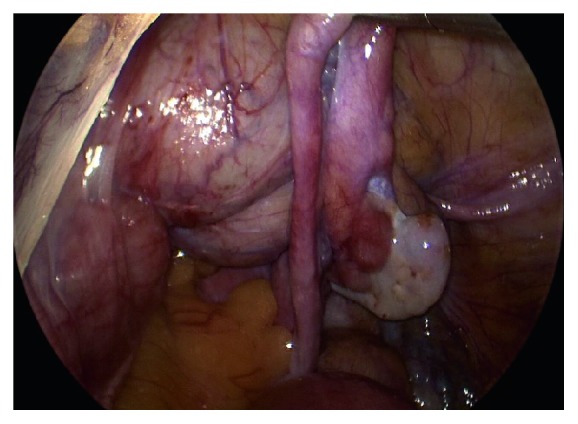
The tube is elevated and the fimbriae and ovary are seen to the right of the isthmus of the tube. The uterine fundus is just visible under a layer of bowel fat.

When the fundus of the uterus was exposed, both of the fallopian tubes were identified and traced to their termination at the fimbriae for confirmation. The isthmus of the tube was elevated with a grasping forcep, and a bipolar vessel sealing device was used to seal and divide a 1-2 cm segment of the tube from its attachments to the rest of the tube and the mesosalpinx using a modified laparoscopic Pomery method. See Figure 5. The excised segment of tube was removed from the abdomen through an accessory port. The pedicles were inspected to ensure hemostasis. The procedure was then repeated on the contralateral fallopian tube without complications.

At the completion of the procedure, the cannulas were then withdrawn under direct visualization to prevent inadvertent herniation and ensure hemostasis. The gas was released from the abdomen. The fascia at all four port sites was then closed with an interrupted cruciate using 0 PDS suture. The skin and subcutaneous tissue were closed with a 4-0 monofilament suture in a subcuticular pattern. Incisions were dressed with skin glue. Several additional drops of skin glue were placed around the animal's skin as a distraction from picking at the actual incisions in the early postoperative period.

The chimp was then transferred back to her cage, extubated, and monitored. Following the procedure, her postoperative recovery was without complications. She was given a single dose of long-acting ceftiofur (20 mg/kg subcutaneous injection) for antibiotic prophylaxis, as there was no guarantee of keeping her or the incisions clean in the zoo. She was visibly uncomfortable the day following the procedure, and was treated with meloxicam (0.14 mg/kg oral tablet daily for 7 days) and tramadol (1 mg/kg oral tablet twice daily for 3 days) for pain. She quickly resumed her normal activities thereafter, and by 14 days post-op, she was well-healed and had returned to the group.

## 6. Physiologic Monitoring and Anesthesia

It is important to remember that Trendelenburg positioning and abdominal insufflation with CO_2_ gas puts considerable stress on the animal. Breathing is shallow because of pressure on the diaphragm. Absorption of CO_2_ from the abdomen can cause acidosis and consequent arrhythmias can occur. To avoid these effects, natural respiration should be continuously supplemented by mechanical ventilation to flush CO_2_ out of the system. After insertion of trocars, abdominal insufflation should be reduced to the minimum necessary amount to permit visualization of the internal genitalia. In animals in general, optimal intraperitoneal pressure is between 7 and 8 mmHg if it provides adequate visualization. This pressure is used rather than using higher pressures (10–15 mmHg) in order to minimize the possible effects on cardiovascular parameters and splanchnic blood flow [5, 6].

## 7. Discussion

Laparoscopy offers a minimally invasive approach to pelvic and intra-abdominal surgery and carries advantages over traditional open surgery. Shorter recovery times are preferable, as the concepts of “rest” and “activity restriction” necessary for proper healing are easily lost on animals after the initial discomfort has faded. This could easily lead to a fascial dehiscence, hernia, or surgical site infection, all of which can carry substantial morbidity. While there is still a small risk of port-site hernia with laparoscopic surgery, this risk is nearly negligible in 5 mm ports [7]. Ensuring a fascial closure stitch is placed in any 10 mm or greater port sites drastically reduces the risk of hernia [7]. As laparoscopic technology advances and 3 mm “micro-laparoscopy” instruments become more widely available, surgeons may be able to eliminate the need for a 10 mm port all together.

In addition to decreased complications, fewer postoperative infections, and shorter recovery times, laparoscopy has gained a lot of momentum due to overall cost savings [8]. One determinant of cost-savings for laparoscopy in humans is the reduced length of hospital stay. For example, patients will often be discharged the same day following a laparoscopic hysterectomy, and rarely require overnight observation in routine cases. This is in comparison to a 2–4 day hospitalization for traditional open hysterectomy. Studies show that this is also true in several different species including dogs. One study monitored the activity of dogs who had undergone surgery using activity trackers on their collars. At 24 hours post-op, there was a 62% decrease in activity counts in animals who had open ovariectomy, versus only a 25% decrease in activity counts in animals who underwent laparoscopic ovariectomy [9].

The technique described does require a team effort including surgeons, circulating nurse, anesthesiologist, scrub tech, and other ancillary support. In this case, a team of human minimally invasive gynecologic surgeons served as consultants given the similar anatomy to humans. With the right training, this procedure could be performed by a veterinary laparoscopic surgeon. A scrub tech was also used to streamline the procedure and avoid delays causing longer time under anesthesia for the animal. They can also serve as an extra pair of hands for retraction and manipulation of the uterus as well.

In this case, one hurdle that was encountered was gaining adequate visualization of the uterus and fallopian tubes. Intra-abdominal adhesions were encountered despite no history of prior abdominal surgery. While it is unclear why these adhesions were present, one theory is that it could be due to small foci of inflammatory reactions secondary to “micro-perforations” in the gastrointestinal tract caused by sharp objects ingested as part of the primates diet. This could theoretically cause adhesions in a similar process as diverticulitis.

In addition to intra-abdominal adhesions, the cephalad and intra-abdominal position of the bladder causes it to protrude into the surgical field if not adequately drained. The ascending and descending colon tend to lose their peritoneal supports, and the transverse mesocolon is long and well-developed and drapes into the surgical field. This not only obscures the visualization for the surgeon, but also puts these vital organs at risk of injury. In addition, some primates such as gorillas tend to have a very tubular pelvic canal compared to the bowl-shaped pelvis of the female human. This may also make access to deep pelvic structures challenging, though not impossible. With well-placed retraction instruments and a uterine manipulator to elevate the uterus up out of the pelvis, we were able to successfully complete this procedure. However, if the anatomy and/or adhesions prevent the surgeon from being able to safely proceed, the procedure should be converted to an open laparotomy approach.

Despite these hurdles, laparoscopic sterilization was successfully completed on this chimpanzee. The approach to laparoscopy described here can be adopted to a wider range of surgical procedures as well in the pelvis and abdominal cavity. This will allow for more species to be able to benefit from the advantages of laparoscopic surgery.

## Figures and Tables

**Table 1:** List of equipment used during the procedure.

Laparoscope, 5 mm, 0 degree angle
Automatic insufflator
CO_2_ cylinder
Light projector and fiber light cable
LCD visual display screen
Cannulas with trocars, 5 mm × 3
Blunt grasping forcep, 5 mm
Weiner grasping forcep, 5 mm
Fanning bowel retractor, 5 mm
Bipolar vessel sealing device
Tilting operating table
Electrocautery unit
Emergency laparotomy tray
Ring forceps
Speculum, small
